# Clinical Cases

**Published:** 1890-11

**Authors:** Edward Rushmore

**Affiliations:** Plainfield, N. J.; Clinical Bureau, I. H. A.


### CLINICAL CASES.

EDWARD RUSHMORE, M. D., PLAINFIELD, N. J.

(Clinical Bureau, I. H. A.)

INTERMITTENT FEVER—SEPIA.—A middle-aged, single lady, living near swampy ground, has chilliness in the afternoon, with coldness of the nose. She wakens at three A. M., with heat and throbbing in the abdomen, followed with perspiration of the feet and of the palms. Several other members of the family have had intermittents in former years. Sepia$^{cm}$ (F.), one dose dry, and subsequently repeated in water, cured her.

PROLAPSE OF RECTUM. BACKACHE—RUTA, CONIUM.—An old gentleman had prolapse of the rectum preceding very difficult stool. Ruta$^{900}$ (F.), one dose, entirely relieved him of this difficulty. On another occasion, the same gentleman complained of a dribbling, intermittent flow while urinating. He received one dose of Conium$^{900}$ (F.), and said it fully relieved him.

Of Ruta, I would like to add that, in the early stage of a malignant disease of kidney and bladder, it relieved, for a considerable time the pain in the back, and was given because the pain in the back was relieved by lying down on the back. This condition has been verified as a trustworthy indication for the use of Ruta in many other cases.

COUGH—KALI-CARBONICUM.—A boy of eleven years has grown very fast; he gets an ulcerated sore throat from slight exposure, and has often had a cough. A brother died before this patient was born from scrofulous swellings following scarlatina, under the treatment of an eclectic homœopath. He now has a severe cough day and night, with vomiting of food. The cough is worse about three to four o'clock in the morning. He sweats easily. The pulse is one hundred and fourteen. Temperature one hundred and one to one hundred and two. There is sharp, bronchial respiration in the upper part of the right lung posteriorly. Loss of appetite and of flesh as well. He has been under the care of the same eclectic homœopath.

He received one dose of Kali-carbonicum$^{cm}$ (F.), graft. At the end of three weeks, the pulse and temperature were normal, and the cough almost wholly removed.

Melancholy—Sulphur.—A young lady, scarcely grown, has, for a long time, suffered from melancholy and depression, a feeling as if she is going to die. She is dizzy and faint on rising in the morning, and has occasional obscuration of sight, when objects appear both dim and crooked, and it seems as if her eyes are not in their proper place. Besides, she has a bad taste in the mouth, weakness at the stomach, and eructations if she takes coffee, and constipation (if she takes coffee), for which she has taken Aloes. The menses occur four or five days too soon, but are otherwise natural. She is sleepy by day, more so in the afternoon, but sleeps well at night. She is easily chilled, and takes cold easily. To get the feet the least damp makes the forehead feel heavy, the eyes feel strange, and gives them a hollow look.

She received one dose of Sulphur$^{cm}$ (F. C.). Six months later her father, visiting me for himself, told me she recovered both from the melancholy and the bodily ailments soon after taking the medicine, and that she had remained well to that time. I have every reason to think that she has had no return of the symptoms.

Uterine Bearing Down—Belladonna.—An aged lady complained of a sense of fullness and much bearing down in the lower abdomen, worse in lying down, and relieved by sitting up. Belladonna$^{50m}$ (F. C.) relieved her in a short time.

Heart Coldness and Trembling—Natrum-muriaticum.—An aged lady had a sense of coldness at the precordia, and trembling of the heart. Natrum-muriaticum$^{900}$ (F.), one dose, removed both these symptoms.

Chronic Cough—Ammonium-muriaticum.—A gentleman wrote me from California that he had suffered there, for several years, with bronchitis and cough, with a feeling of coldness between the scapulæ. I sent him Amm-mur.$^{20m}$ (F.), ten powders, to be taken at intervals. In about three months he wrote to say that he was so much better he thought he did not need more medicine.

I always examine Amm-mur. in cases of cough, with coldness between scapulæ or shoulders. It has nearly always been found suitable and helpful.

---

